# P16 The wood for the trees

**DOI:** 10.1093/rap/rkad070.037

**Published:** 2023-09-27

**Authors:** Sadia Tareq, Edward Tranah, Kevin Tsoi

**Affiliations:** Epsom and St.Helier University Hospitals, Sutton, United Kingdom; Epsom and St.Helier University Hospital, Sutton, United Kingdom; Epsom and St.Helier University Hospital, Sutton, United Kingdom

## Abstract

**Introduction:**

Knowing when to screen for malignancy is an important aspect of care within rheumatology. We herein present the case of a patient with rheumatoid arthritis who developed painful and circumferential swelling in both lower limbs which rapidly progressed to involve her arms, trunk and abdomen, following a third cycle of rituximab. On examination, her skin was indurated and ‘woody’ with the Groove sign and peau d’orange skin changes apparent in the upper limbs. Blood tests revealed eosinophilia. Will you recognise the trees when you see this patient next? And if you do, will you see the wood?

**Case description:**

A 60-year-old Caucasian woman, already known to our rheumatology department with seropositive rheumatoid arthritis, was seen between scheduled appointments with the development of painful swelling of both lower limbs following her 3rd cycle of rituximab. Past medical history was also significant for breast cancer, osteoporosis and depression. Medications included alendronic acid and escitalopram. Rituximab had been commenced after intolerances to methotrexate, hydroxychloroquine and leflunomide. She was a non-smoker and her family history was significant for breast cancer.

On examination there was symmetrical, circumferential non-pitting oedema of all limbs with thickened ‘woody’ skin and induration across the lower trunk and upper abdomen. Venous guttering and a peau d’orange appearance was present in the upper limbs alongside fixed extension deformities of both elbows. The hands, feet and face were spared.

Blood tests revealed a white cell count of 12.6 x 109/L with eosinophilia 2.5 x109/L, ESR 25 mm/hr and C-reactive protein 74 mg/L. ANA & ENA screens were negative.

A clinical diagnosis of eosinophilic fasciitis was made and a course of prednisolone 30 mg once daily was started with marked clinical improvement. MRI of the lower limbs demonstrated widespread subcutaneous oedema and fascial thickening. PET-CT identified a small area of subtly increased tracer uptake in the liver with no abnormal soft tissue or specific increased activity within either breast. Painful swelling recurred at a dose of prednisolone 5 mg which prompted a return to a higher dose with a slower wean.

Ultrasound-guided biopsy confirmed a diagnosis of stage IV relapsed breast cancer. Treatment with exemestane and palbociclib has been initiated but our patient has since been hospitalised with suspected sepsis. Median survival is 5 years and there needs to be a frank discussion regarding quality of life on long-term low dose steroids versus re-introduction of methotrexate with increased risk of cytopaenias when used concurrently with palbociclib.

**Discussion:**

Once a diagnosis of eosinophilic fasciitis (EF) was made, we pursued PET-CT imaging to assess for malignancy. This was felt to be of particular importance in this case in view of the personal history of breast cancer and a knowledge of the potential for a paraneoplastic aetiology. The decision to assess for malignancy in this way is arguably the most important decision we have made to date for our patient.

Following discussions with the patient’s dermatology and oncology teams, and prior to our knowledge of hospital admission with suspected sepsis, we had decided to recommend the commencement of a steroid-sparing agent to our patient. Methotrexate was our initial choice over mycophenolate mofetil (MMF) to maintain remission as not only will it treat her rheumatoid arthritis but it is also carries a lower risk of myelosuppression. Furthermore, no cytopaenias were induced when our patient was previously on methotrexate.

Not only have we learned about the diagnostic delay that so frequently affects those with EF and the substantial morbidity that it confers, we have learned that the management of EF is not standardised and requires a multi-disciplinary approach which may include, but is certainly not limited to, the involvement of dermatology, radiology, oncology, general surgery where fascial biopsy is pursued, and allied health professionals.

Our case is interesting because it highlights the typical presentation of a little-known condition with potentially life-threatening associations that, in this case, was diagnosed in the rheumatology outpatient department. We are interested to hear what our colleagues might do next in the management of this case. In the context of serious infection whilst receiving chemotherapy, the additional risk of cytopaenias with methotrexate and an overall poor prognosis, would delegates consider management solely with long-term glucocorticoids in this instance?

**Key learning points:**

Our main learning points, thus far, are as follows:

Eosinophilic fasciitis (EF) was first discovered and described by Dr Lawrence E. Shulman in 1975 and was previously known as Shulman syndrome. It is a rare disorder characterised by fascial thickening with an eosinophilic tissue infiltrate and peripheral eosinophilia. Clinical presentation is with subacute onset of symmetrical, circumferential swelling of the limbs with tight, thickened skin, pain and joint restriction. Typical examination findings include ‘woody’ induration of the skin and other changes such as the Groove sign (venous guttering as a result of perivenular fat loss) and peau d’orange with sparing of the hands, face and feet.

Screening for malignancy should aways be considered. Approximately 10% of cases of EF are thought to be paraneoplastic; an underlying haematological malignancy is most common, with breast and lung cancers being the most frequently implicated solid malignancies.

The differential diagnosis of EF is broad and includes morphoea, systemic sclerosis and infiltrative skin conditions such as amyloidosis. Relevant history might be suggestive of other conditions such as sclerodermoid graft-versus-host disease (GvHD) or nephrogenic systemic fibrosis. Scleredema and scleromyxoedema are also differential diagnoses.

Histology can be helpful but is not mandatory in the diagnosis of EF.

The cornerstone of remission induction is glucocorticoids. Remission maintenance is typically achieved with methotrexate, mycophenolate mofetil (MMF) or azathioprine. There exist a number of case reports describing the use of infliximab, rituximab and tocilizumab in refractory cases of EF.

Abatacept is licensed for use in cases of severe treatment-resistant localised scleroderma which can occur in the later stages of fasciitis condition such as EF.

At the conference we would like to gain insight on the pathogenesis of EF, alternative management strategies that we may not have considered, and ideas on what next steps might benefit our patient the most.

